# Navigating electronic health record accuracy by examination of sex incongruent conditions

**DOI:** 10.1093/jamia/ocae236

**Published:** 2024-09-10

**Authors:** Ling Cai, Ralph J DeBerardinis, Xiaowei Zhan, Guanghua Xiao, Yang Xie

**Affiliations:** Quantitative Biomedical Research Center, Peter O’Donnell Jr. School of Public Health, University of Texas Southwestern Medical Center, Dallas, TX 75390, United States; Children’s Research Institute, University of Texas Southwestern Medical Center, Dallas, TX 75390, United States; Simmons Comprehensive Cancer Center, University of Texas Southwestern Medical Center, Dallas, TX 75390, United States; Children’s Research Institute, University of Texas Southwestern Medical Center, Dallas, TX 75390, United States; Simmons Comprehensive Cancer Center, University of Texas Southwestern Medical Center, Dallas, TX 75390, United States; Howard Hughes Medical Institute, University of Texas Southwestern Medical Center, Dallas, TX 75390, United States; Quantitative Biomedical Research Center, Peter O’Donnell Jr. School of Public Health, University of Texas Southwestern Medical Center, Dallas, TX 75390, United States; Simmons Comprehensive Cancer Center, University of Texas Southwestern Medical Center, Dallas, TX 75390, United States; Center for Genetics of Host Defense, University of Texas Southwestern Medical Center, Dallas, TX 75390, United States; Quantitative Biomedical Research Center, Peter O’Donnell Jr. School of Public Health, University of Texas Southwestern Medical Center, Dallas, TX 75390, United States; Simmons Comprehensive Cancer Center, University of Texas Southwestern Medical Center, Dallas, TX 75390, United States; Department of Bioinformatics, University of Texas Southwestern Medical Center, Dallas, TX 75390, United States; Quantitative Biomedical Research Center, Peter O’Donnell Jr. School of Public Health, University of Texas Southwestern Medical Center, Dallas, TX 75390, United States; Simmons Comprehensive Cancer Center, University of Texas Southwestern Medical Center, Dallas, TX 75390, United States; Department of Bioinformatics, University of Texas Southwestern Medical Center, Dallas, TX 75390, United States

**Keywords:** electronic health records, quality assessment, sex, disconcordant, *All of Us*

## Abstract

**Objective:**

The increasing reliance on electronic health records (EHRs) for research and clinical care necessitates robust methods for assessing data quality and identifying inconsistencies. To address this need, we develop and apply the incongruence rate (IR) using sex-specific medical conditions. We also characterized participants with incongruent records to better understand the scope and nature of data discrepancies.

**Materials and Methods:**

In this cross-sectional study, we used the *All of Us* Research Program’s latest version 7 (v7) EHR data to identify prevalent sex-specific conditions and evaluated the occurrence of incongruent cases, quantified as IR.

**Results:**

Among the 92 597 males and 152 551 females with condition occurrence data available from *All of Us* and sex-conformed gender, we identified 167 prevalent sex-specific conditions. Among the 37 537 biological males and 95 499 biological females with these sex-specific conditions, we detected an overall IR of 0.86%. Attempt to include non-cisgender participants result in inflated overall IR. Additionally, a significant proportion of participants with incongruent conditions also presented with conditions congruent to their biological sex, indicating a mix of accurate and erroneous records. These incongruences were not geographically or temporally isolated, suggesting systematic issues in EHR data integrity.

**Discussion:**

Our findings call attention to the existence of systemic data incongruences in sex-specific conditions and the need for robust validation checks. Extending IR evaluation to non-cisgender participants or non-sex-based conditions remain a challenge.

**Conclusion:**

The sex condition-specific IR, when applied to adult populations, provides a valuable metric for data quality assessment in EHRs.

## Introduction

In the era of big data, the integrity of electronic health records (EHRs) is the cornerstone of modern medical research and personalized healthcare. As the volume and complexity of health data grow so does the difficulty of validating these datasets. This is primarily due to the diverse sources and methods of data entry, which can lead to inconsistencies and errors.^1^^,^^2^ As reviewed by Callahan et al,^3^ there have been long-standing concerns around the quality of EHR-generated data,^4–8^ considerable efforts have been undertaken to develop methods for data validation.^1^^,^^9–12^ Identifying logical and scalable methods to confirm the fidelity of EHR data remains a top priority in the field. Professional organizations, including the American Medical Informatics Association, have emphasized the need for robust data validation processes and recommend the development of advanced algorithms and frameworks that can automatically detect and correct discrepancies in large datasets.^13^ The data quality assessment (DQA) framework proposed by Kahn et al^14^ highlight conformance, completeness, and plausibility. Following this framework, comprehensive rule-based DQA is being established, with demonstrated utility in a single-center case study.^15^

Under the DQA framework, plausibility assesses whether the values, distributions, or densities observed in data conform to locally accepted knowledge (for verification) or align with trusted external references or established standards (for validation).^14^ Sex-based checks in adult studies stand out as a practical and revealing approach to estimate data correctness by atemporal plausibility and concordance.^14^ In this context, plausibility examines whether the health conditions recorded are biologically or medically possible for the recorded sex, while concordance evaluates the agreement between medical conditions, survey responses and genomics-inferred sex, with genomics-inferred sex regarded as the gold standard.

The National Institutes of Health launched the *All of Us* Research Program with the ambitious goal of revolutionizing precision medicine.^16^ This initiative is unique not only in its scale—aiming to gather comprehensive health data from over 1 million participants across the United States—but also in its commitment to diversity and inclusion, seeking to include participants from a myriad of backgrounds, including underrepresented racial and ethnic groups, age brackets, geographic regions, and health statuses. By analyzing a broad spectrum of data types, including genomics data, environmental lifestyle information from survey data and clinical data from EHR, the program aims to uncover patterns that may help predict the onset of diseases and the effectiveness of treatments for individual patients. Maintaining the integrity of such extensive health databases is crucial for deriving accurate, personalized medical insights.

In this study, we take the latest version 7 (v7) of the *All of Us* data and use a data-driven approach to identify sex-specific conditions that could be used for sex-based checks. We use incongruence rate (IR) to assess concordance between sex-specific medical conditions and the self-reported sex data, supported by orthogonal genomic data. We further characterize participants with incongruent records to better understand the scope and nature of data discrepancies. By doing so, we highlight areas that require attention and hope to contribute to the improvement of the program’s data curation process and the development of robust validation mechanisms for large-scale health databases in general.

## Methods

### Study cohort and data source

This cross-sectional analysis utilized the *All of Us* controlled tier dataset version 7 (v7), covering participant enrollment from May 31, 2017, to July 1, 2022. Enrollment in the *All of Us* program is open to all US adults who can provide informed consent and are not incarcerated. Participants engage with the program either online or via partner healthcare provider organizations dispersed across the United States. The University of Texas Southwestern Medical Center institutional review board determined that this research did not constitute human subject research as the *All of Us* data available to researchers are all deidentified, and therefore, waived the need for additional approval and informed consent. As of the v7 data release, over 430 000 participants’ data have been curated, including EHRs, physical measurements, and responses to various questionnaires.

### Data acquisition and analysis

We performed our analyses using the R statistical software (version 4.4.0).^17^ This included the use of packages such as bigrquery_1.5.1^18^ for interfacing with Google’s BigQuery, tidyverse_2.0.0,^19^ lubridate_1.9.3^20^ and data.table_1.15.4^21^ for data manipulation, ggplot2_3.4.4^22^ and patchwork_1.2.0^23^ for visualization. We queried the “person” table for sex at birth (biological sex) and gender (personal sense of gender identity) from survey, the “condition_occurrence” table for EHR-derived condition records and their respective start dates, and the “socioeconomic status” table for participants’ zip codes (with last 2 digits masked). Concept IDs, which are unique identifiers used in the *All of Us* EHR data to standardize medical data elements in the Observational Medical Outcomes Partnership (OMOP) Common Data Model, were interpreted with the OMOP Standardized Vocabularies v5.0. Additionally, we extracted genomic-inferred biological sex for the participants with incongruent sex-specific conditions from the genotyping array data (autocallGender field in the vcf files under gs://fc-aou-datasets-controlled/pooled/microarray/vcf/v7_base/).

### Selection of sex-specific conditions

**Figure 1.**
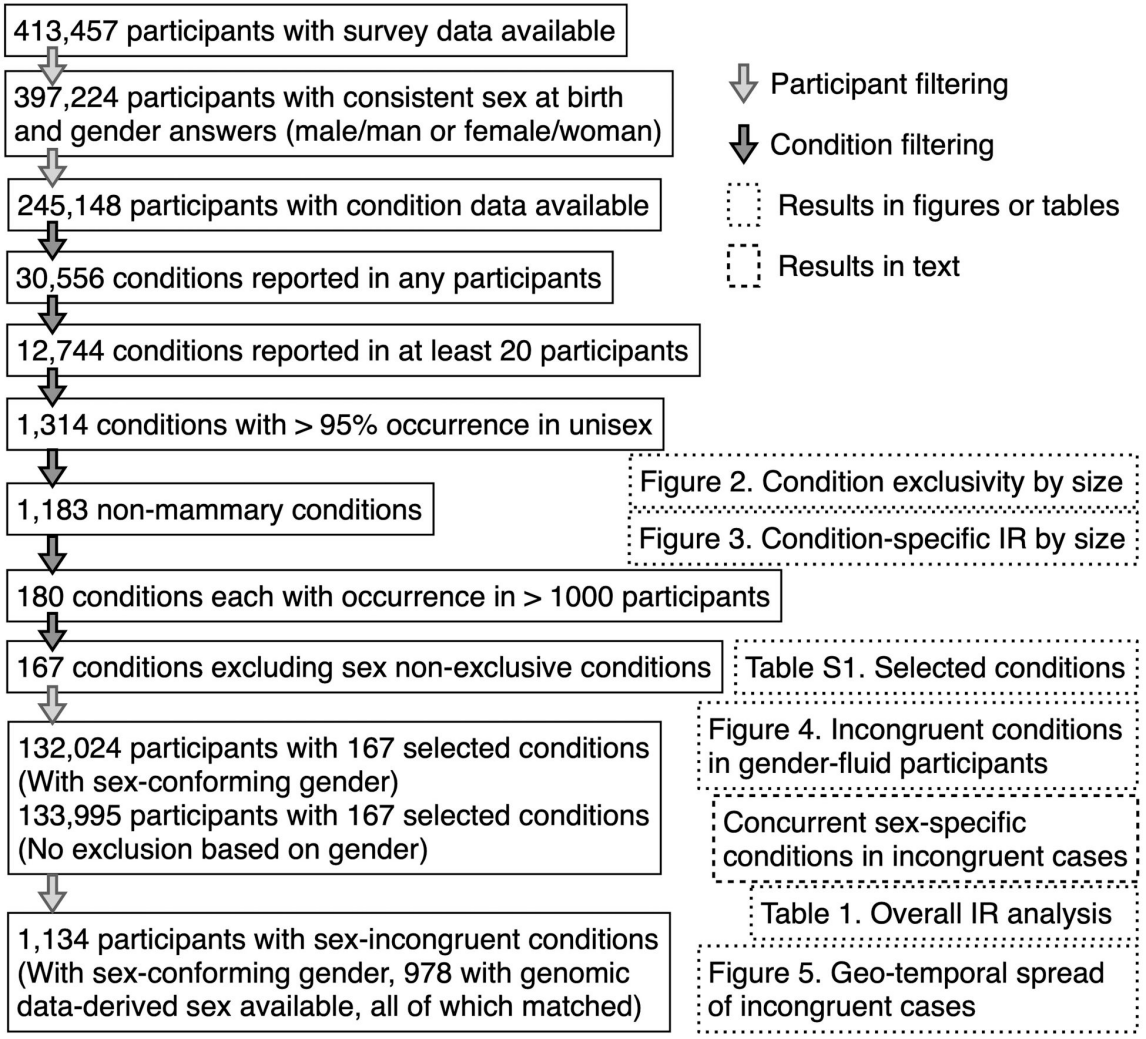
Participant filtering, condition filtering, and analysis overview.

From the 413 457 participants with survey data available, we selected participants with male or female answers as their response to the sex_at_birth survey question and excluded participants with answers other than man and woman in their gender identity survey, to avoid ambiguity in sex-specific condition that may arise from gender affirming surgeries. The 16 233 excluded participants, however, also included those who skipped or preferred not to answer these questions. The condition occurrence data are available for 92 597 male and 152 551 female (245 148 total) cisgender participants. Subsequently, from 30 556 conditions recorded in any of these participants’ EHR, we filtered out conditions that occurred in fewer than 20 participants, leaving 12 744 conditions. We then selected conditions with more than 95% (similar to the 5% *P* value standard in statistical testing) of occurrences found in participants of a single sex, either male or female at birth. Conditions of breast/mammary findings are excluded because although less common, males can also develop such conditions. This resulted in 1183 conditions with strong sex bias. Refer to Figure 1 for our schema as a flowchart.

### IR as a DQA metric

To ascertain the quality of EHRs, we propose the IR as a metric for assessing data integrity. This metric is grounded in the identification of conditions that are biologically implausible given a patient’s characteristics, such as sex and age—attributes that are available from independent surveys or genomic data. In this study, we narrow our focus to sex-specific conditions.

Condition-specific incongruence rate (CIR): For each sex-specific condition, we calculate a CIR. Let $N_{\mathrm{incongruent},\mathrm{cs}}$ be the count of participants with a specific condition that conflicts with their sex recorded at birth, and $N_{\mathrm{total},\mathrm{cs}}$ be the total number of participants diagnosed with that condition. Thus, for each condition, we have


$$\mathrm{CIR}=\frac{N_{\mathrm{incongruent},\mathrm{cs}}}{N_{\mathrm{total},\mathrm{cs}}}.$$

Recognizing CIR can be highly heterogeneous, we recommend treating sex-specific conditions collectively to calculate an overall incongruence rate (OIR): We define $N_{\mathrm{incongruent},\mathrm{overall}}$ as the number of participants with any of the preselected sex-specific conditions but have biological sex incongruent to the condition. $N_{\mathrm{total},\mathrm{overall}}$ represents the count of participants with that set of preselected sex-specific conditions. The OIR is defined as follows:


$$\mathrm{OIR}=\frac{N_{\mathrm{incongruent},\mathrm{overall}}}{N_{\mathrm{total},\mathrm{overall}}}.$$

The IR provides a direct measure of the level of data incongruity within the dataset. A higher IR indicates a greater proportion of data inconsistency, which may signal the need for review or corrective measures in data curation.

As IR is a proportion, we also provide the confidence interval (CI) for IR as follows:


$$\mathrm{CI}=p\pm z\times \sqrt{\frac{p(1-p)}{n}},$$

where *p* is the proportion, in our case, IR; *n* is the total number of participants with the condition; *z* is the *z*-score corresponding to the desired confidence level (1.96 for 95% CI).

### Examination of IR in sex-specific conditions by total participant counts

**Figure 2.**
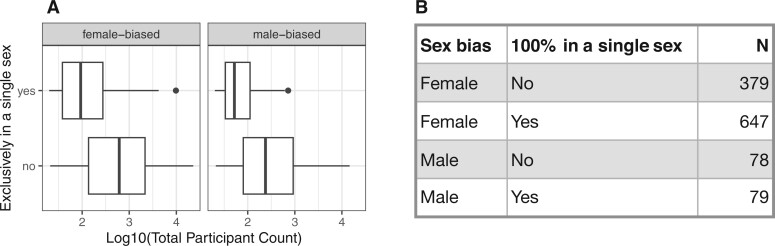
Sex-biased conditions found in 100% single-sex participants are smaller in size. (A) Total participant count per condition (*x*-axis) is plotted against concordance (*y*-axis), separated into panels for female-biased and male-biased conditions. (B) A summary table showing the number of conditions with and without perfect concordance (100% in a single sex) to the expected sex bias. Note that conditions with a female bias are more prevalent than those with a male bias.

We applied a log transformation (log10) to the total participant count to stabilize variance and normalize the data distribution, which varies widely across conditions. This method enhances data interpretability in our scatterplots in Figure 2. In refining our analysis, we concentrated on conditions reported in 1000 or more participants to minimize potential bias from low-frequency conditions, which may inflate perceived IRs, thus providing a more robust measure of data issues (refer to Figure 2B). This decision was based on preliminary observations that lower total participant counts could distort the reliability of sex-specific IRs, as shown in Figure 2A. In light of conditions like hirsutism exhibiting sex bias but still affecting both sexes, we utilized ChatGPT to assist in manually reviewing 180 conditions that exhibit sex bias but affect both sexes, a process that did not involve human subjects or identifiable private information, and thus did not require IRB approval. For clarity and reproducibility, the specific version of ChatGPT used in this study was ChatGPT-4, with the 180-condition concept name entered and the following prompt: “Here is a list of conditions. Please go through all of these terms and provide justification one at a time, explain whether they are sex-specific based on your knowledge. At the end, construct the list of non-sex-specific conditions in the form of an R character vector.” A physician manually went through these conditions to correct those misclassified.

## Results

### Selection of sex-specific conditions

*All of Us*’ v7 curated data repository houses EHR data from over 287 000 participants. Our initial analyses of a sex-specific condition incidentally revealed the surprising presence of participants of the opposite sex. These instances prompted us to conduct a thorough methodological review outlined in Figure 1. We refined our assessment approach by analyzing sex-specific conditions screened from the condition occurrence database (see Methods). To avoid ambiguity from non-cisgender participants, we only included those with sex-conformed gender and identified 1183 conditions with strong sex bias. The most prevalent female-biased condition is “Finding related to pregnancy,” with 209 males and 22 625 females. Conversely, the most prevalent male-biased condition is “Primary malignant neoplasm of prostate,” with less than 20 females and 14 582 males. We observed that conditions with 100% occurrence in a single sex tended to have lower total participant counts (Figure 2A). This suggests that smaller sample sizes could obscure the recognition of discordant cases. Notably, the number of conditions predominantly affecting females significantly exceeded those affecting males (Figure 2B).

**Figure 3.**
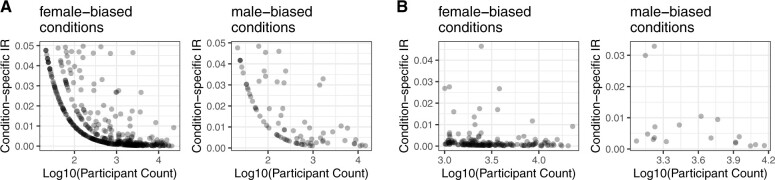
Incongruence rates in sex-biased conditions by total participant count. (A) Scatter plot showing the relationship between the log-transformed total participant count for each condition (*x*-axis) and the condition-specific incongruence rate, CIR (*y*-axis), where CIR refers to the proportion of subjects whose sex does not align with the expected majority for sex-specific conditions. The left panel depicts conditions typically associated with females (female conditions), and the right panel depicts those typically associated with males (male conditions). The observed pattern indicates that conditions with higher total participant counts have a CIR, suggesting increased data reliability for these conditions. (B) Focused view of the conditions with higher total participant counts (>1000), which are more pertinent for assessing the validity of sex-specific prevalence.

Given that lower total participant counts may artificially inflate the perceived IRs and therefore provide a less reliable measure of this data issue (Figure 3A), our analysis was refined to concentrate on conditions reported in 1000 or more participants (Figure 3B). We also removed 12 non-exclusive conditions after manual review (see Methods). From this point on, we term these sex-specific conditions instead of sex-biased conditions. The full list of 167 sex-specific conditions is provided in Table S1 with their OMOP concept IDs.

### IRs for sex-specific conditions

To assess data quality quantitatively, we introduce the metric IR (see Methods), which includes CIR and OIR. From the 167 selected conditions, there are 151 female-specific conditions and 16 male-specific conditions. The top 3 conditions from both sexes with the highest frequency of sex-incongruent participants are listed in the top part of Table 1. The counts and frequencies of the 167 conditions are summarized in Table S1. We provide complete data for 17 conditions with at least 20 participants in either sex, whereas the counts and frequencies for the remaining conditions are masked to abide by the *All of Us* data policy to not report numbers for groups with fewer than 20 individuals.

**Table 1.** Top conditions with the highest condition-specific IR and overall IR.

Sex specificity	OMOP ID	Condition	Incongruence count	Congruence count	Condition-specific IR	Confidence interval
Female	440787	Drug dependence in mother complicating pregnancy, childbirth, AND/OR puerperium	27	1005	0.0269	[−0.034, 0.088]
Female	201909	Female infertility	102	3715	0.0267	[−0.0046, 0.058]
Female	4279913	Primary ovarian failure	62	3591	0.0170	[−0.015, 0.049]
Male	197605	Inflammatory disorder of male genital organ	42	1361	0.0299	[−0.022, 0.081]
Male	26662	Testicular hypofunction	44	4157	0.0105	[−0.02, 0.041]
Male	200962	Primary malignant neoplasm of prostate	55	5770	0.00944	[−0.016, 0.035]

**Collective conditions**			**Incongruence count**	**Congruence count**	**Overall IR**	**Confidence interval**

151 female-specific conditions			849	95 015	0.00886	[0.0083, 0.0094]
16 male-specific conditions			285	35 875	0.00788	[0.007, 0.0088]
167 female or male-specific conditions			1134	130 890	0.00859	[0.0081, 0.0091]

Abbreviations: IR = incongruence rate; OMOP = Observational Medical Outcomes Partnership.

As the CIRs are highly variable for individual conditions with CIs spanning zero, we decided to treat the sex-specific conditions collectively to calculate the OIR. We identified 849 males and 95 015 females with any of the 151 female-specific conditions. For the 16 male-specific conditions, there were 285 females and 35 875 males. We were able to retrieve the genomics data-inferred sex for 978 of these 1134 participants with incongruent conditions and saw a perfect match with their sex at birth, confirming the reliability of the survey data. The combined OIR is 0.86% with OIRs for female-specific (0.89%) and male-specific (0.79%) conditions (Table 1, bottom).

### Inclusion of non-cisgender participants may overestimate OIR

**Figure 4.**
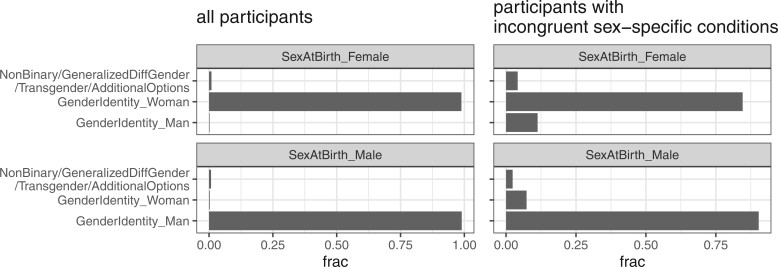
Non-cisgender participants may inflate incongruent cases when included. Gender identity fractions in all participants are based on 404 734 participants with sex at birth being either male or female. Gender identity fractions for participants with incongruent sex-specific conditions are based on 1288 participants. Note that subjects with “Skip” or “PreferNotToAnswer” have been removed from this analysis.

We also computed OIRs with the inclusion of participants without sex-conforming gender (including those with gender opposite to sex at birth, transgender, non-binary, and skipped answers), and found an inflated OIR of 0.96% [0.91%, 1.00%], implying higher rates of incongruent case in the non-cisgender population. In the v7 *All of Us* database, for both sexes, those with gender identities different from their sex at birth account for 1.11% of the entire cohort (Figure 4). When included, non-cisgender participants comprise 15% of the subjects where biological females are documented with male-specific conditions and 10% of the subjects, where biological males are documented with female-specific conditions, suggesting that the inclusion of non-cisgender participants may overestimate sex-based IR.

### Concurrent sex-specific conditions in incongruent cases

We next examined the incongruent cases to see if they also have sex-specific conditions congruent to their sexes. Among the 285 females with male-specific conditions, 87% also had female-specific conditions. Similarly, 69% of the 849 males with female-specific conditions also had male-specific conditions. These findings suggest a likely mix of both accurate and erroneous records for the participants with incongruent sex-specific conditions.

Furthermore, we investigated the occurrence of multiple incongruent sex-specific conditions within the same individuals. Among those with incongruent conditions, 6% of females and 20% of males had more than 1 distinct incongruent condition recorded. Although these rates are lower compared to the occurrence of multiple conditions among subjects with congruent conditions (77% in females and 60% in males), the frequency of such recurrent errors remains concerning.

### Geographical spread and temporal distribution of incongruent participants and condition occurrence

**Figure 5.**
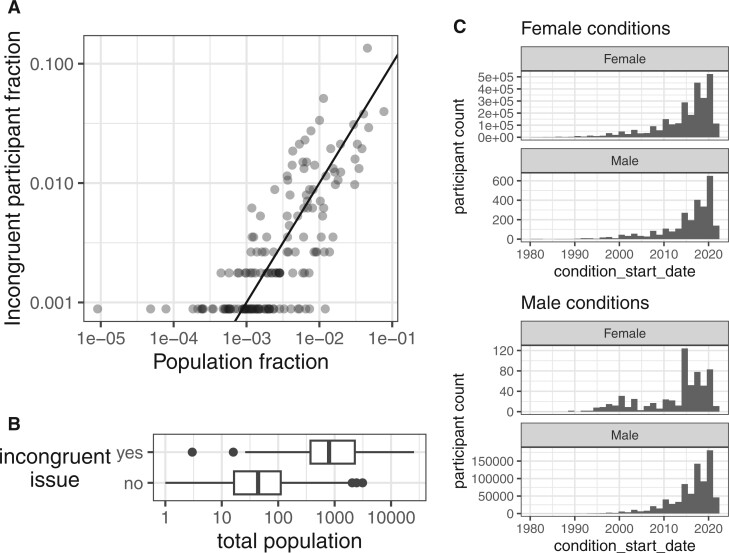
Incongruent cases across zip codes and condition start date. (A) Issue occurrence frequencies correlate with population sizes in geographical locations identified by zip codes. Each dot represents a zip code, with the fraction of the total population on the *x*-axis and the fraction of the participants with incongruent sex conditions on the *y*-axis. Solid line represents *x* = *y*. (B) Comparison of total population sizes between zip codes with and without reported sex-incongruent condition issues. Boxplots differentiate between the 153 zip codes with participants that have incongruent sex-specific condition issues (yes) and the remaining 716 zip codes without issues (no), indicating that zip codes with issues tend to have larger populations. (C) Histograms of condition start year for female-specific conditions (upper panel) and male-specific conditions (lower panel). Patterns of this date distribution are highly similar in both sexes.

To investigate whether this observation was caused by data curation issues from specific Health Provider Organizations, we examined the geographical distribution of the 1134 participants with incongruent sex-specific conditions by extracting their truncated zip codes from the “socioeconomic status” table. These incongruent participants reside in a total of 153 zip codes, the count of the incongruent participants in each zip code highly correlated with the population size (i.e., number of unique subjects of the zip code). As the numbers of participants in each zip code are often less than 20, to abide by the *All of Us* data policy, we visualize fractions instead of the actual count (Figure 5A). The number of incongruent participants within a zip code divided by the sum of all incongruent participants is very close to the population of that zip code divided by the total population across the same set of zip codes. The zip codes without incongruent participants are less populous than those with such issues (Figure 5B), as the limited sample size could obscure such findings. These results suggest that the occurrence of data inconsistencies is not concentrated in specific areas but rather mirrors the population size across regions and the occurrence frequency of the data issue is similar across the country. To determine if the curation issue occurred during a specific time period, we analyzed the start date of conditions in participants with congruent and incongruent sex-specific conditions (Figure 5C). However, the condition onset patterns are highly similar among all groups, indicating that the curation issue is consistent over time, rather than isolated to a specific period.

## Discussion

As the *All of Us* Research Program approaches its goal of enrolling 1 million participants, researchers are presented with unprecedented opportunities to utilize EHR data for community-focused health improvements. However, the integrity of this data should not be assumed. In this study, we highlight potential data quality issues within the *All of Us* dataset and develop methodologies to critically assess its reliability. By applying sex-based quality assessment, we uncovered a systematic OIR of 0.86% within the EHR concerning sex-specific conditions. Despite the large difference in condition numbers and total participant counts for female-specific and male-specific conditions, we observed OIRs in males (0.79%) and females (0.89%), highlighting its utility as a stable measure of data reliability of sex-specific conditions. Importantly, we found that sex at birth data from surveys aligns well with biological sex as determined by microarray genotyping, affirming the reliability of both survey and genomic data, suggesting that the issues we have identified likely stem from EHR data logging or extraction processes rather than participant self-reporting errors in the survey data. This study not only raises awareness among researchers about potential data pitfalls but also enhances their competencies in using the *All of Us* Researcher Workbench for rigorous data evaluation by providing a tangible methodology.

Our strategic filtering led to the refinement of 167 sex-specific conditions, as detailed in Table S1. This curated list serves as a valuable tool for researchers, providing a benchmark for evaluating data integrity across various datasets, perhaps for the *All of Us* data team to incorporate into their standard DQA workflow as well.^24^ Among this collective set of conditions, 69%-87% of the participants with incongruent conditions also had concurrent conditions of both sexes, suggesting a mix of correct and erroneous entries in their EHRs. In analyzing the geographical spread of incongruent sex-specific cases across 153 zip codes, the distribution mirrored local population sizes, suggesting that data curation issues are systemic across the Health Provider Organizations within the *All of Us* database and not isolated to specific regions. Moreover, the consistent occurrence of these issues over time indicates they are likely rooted in long-standing data entry or curation practices or extraction workflow rather than being transient errors.

Interestingly, when non-cisgender participants were included, a higher proportion (10%-15%) of them were found among participants with conditions incongruent to their biological sex, resulting in inflated OIR. While these may be attributed to true diagnosis from gender-affirming surgeries, exclusion of non-cisgender participants in sex-based EHR checking may miss true errors in their data. We examined this subset of non-cisgender individuals, and recognized certain reported conditions remain biologically implausible, such as pregnancies in transgender women and prostate cancer in transgender men.

Sex-based EHR data discrepancies can skew clinical insights for studies of sex-specific conditions. These errors also underscore the potential for broader data quality issues within EHR data logging or extraction workflow. Given that the coding practices for sex-specific conditions are similar to those for non-sex-specific conditions, it is reasonable to speculate that the same systematic errors affecting the former could impact the latter. Furthermore, if we consider the possibility that these incongruent cases arise from random EHR logging errors, such errors are likely more detectable when they involve sex-specific conditions. This is because sex-specific errors are more conspicuous due to their direct conflict with known biological attributes. However, since sex-specific conditions do not apply to all participants and each error has only a 50% chance of being incongruent with the participant’s sex, the actual rate of erroneous EHR entries could be much higher than our observations suggest. Therefore, while our findings focus on sex-specific incongruences, they hint at a potentially larger issue of widespread inaccuracies within the EHR data.

Identification of sex condition incongruence highlights potential issues within EHR-derived datasets, but it has limitations. It is important to note that this approach is primarily applicable to adult populations. Pediatric populations, due to the developmental nature of sex characteristics and associated conditions, may require a different analytical framework. There is also a challenge in developing systematic methods to detect similar issues in non-sex-specific conditions. While demographic variables such as race and ethnicity are pivotal in precision medicine, their definitions are not standardized and vary widely, which challenges automated data checks for plausibility and concordance. Although the method used to detect sex-specific conditions could theoretically apply to age- or ethnicity-specific diseases like macular degeneration, osteoporosis, sickle cell anemia, and cystic fibrosis, discrepancies related to atypical age or ethnicity could represent rare but legitimate cases or misdiagnoses rather than errors in EHR data entry or extraction.

Furthermore, it is imperative to understand whether such data curation disconcordance is unique to the *All of Us* Research Program or if they are indicative of a larger trend within large-scale population studies, including databases like the UK Biobank and the Medical Information Mart for Intensive Care (MIMIC) data sets, or in populations excluded by *All of Us*, such as incarcerated individuals. Comparisons across such repositories could illuminate common challenges and drive improvements in data curation practices.

## Conclusion

As precision medicine continues to evolve, the integrity of the underlying data becomes paramount. Our findings of prevalent sex-incongruent conditions in the *All of Us* EHR-derived data advocate for the implementation of more robust data verification frameworks and highlight the need for ongoing scrutiny of EHR data within large biomedical databases. Moving forward, it is essential that the research community addresses these data curation challenges to ensure the accuracy and utility of health information systems and to foster trust in the insights derived from such significant biomedical research endeavors.

## Supplementary Material

ocae236_Supplementary_Data

## Data Availability

The *All of Us* Research Program’s controlled tier dataset version 7 is accessible through the cloud-based *All of Us* Researcher Workbench, subject to an institutional user agreement. Standardized Vocabularies v5.0, released on August 31, 2023, was downloaded from https://athena.ohdsi.org/. We have deposited our analysis code at https://github.com/cailing20/AoU_sex_incongruent_rates.
