# Supplementary material for: Navigating electronic health record accuracy by examination of sex incongruent conditions
Source: J Am Med Inform Assoc. 2024 Sep 10;31(12):2849–56. doi: 10.1093/jamia/ocae236 (PMC11631140; doi:10.1093/jamia/ocae236)
Supplement: ocae236_Supplementary_Data [file ocae236_supplementary_data.pdf]

Table_S1_167_conditions

sex_specificity	OMOP_id	concept_name	male_count	female_count	total_count	male_frac	female_frac
female	444094	Finding related to pregnancy	209	22625	22834	0.0092	0.9908
female	195867	Noninflammatory disorder of the vagina	<=20	>=20449	20469		
female	4128329	Menopause present	<=20	>=17473	17493		
female	4014295	Single live birth	<=20	>=17026	17046		
female	198194	Female genital organ symptoms	48	15599	15647	0.0031	0.9969
male	198803	Benign prostatic hyperplasia	>=14578	<=20	14598		
female	196168	Irregular periods	<=20	>=14401	14421		
female	4149084	Vaginitis	<=20	>=14186	14206		
female	197610	Cyst of ovary	<=20	>=13611	13631		
female	43530950	Complication occurring during pregnancy	22	12498	12520	0.0018	0.9982
female	197236	Uterine leiomyoma	<=20	>=12337	12357		
male	443211	Benign prostatic hypertrophy with outflow obstruction	>=12260	<=20	12280		
female	4172857	Menopause finding	32	11812	11844	0.0027	0.9973
female	4307820	Unplanned pregnancy	<=20	>=11604	11624		
male	3655355	Erectile dysfunction	>=11090	<=20	11110		
female	4218813	Third trimester pregnancy	<=20	>=10727	10747		
female	4081648	Acute vaginitis	<=20	>=10682	10702		
female	4078455	Finding of menstrual bleeding	<=20	>=10373	10393		
female	4041280	Postpartum finding	<=20	>=9336	9356		
female	443800	Amenorrhea	<=20	>=9170	9190		
female	439082	Menopausal syndrome	29	9103	9132	0.0032	0.9968
female	4188598	High risk pregnancy	<=20	>=8996	9016		
male	43021237	Secondary erectile dysfunction	8871	35	8906	0.9961	0.0039
female	4295261	Postmenopausal state	<=20	>=8711	8731		
female	443431	Disorder of menstruation	<=20	>=8648	8668		
female	439658	Disorder of pregnancy	100	8445	8545	0.0117	0.9883
female	441641	Delivery normal	<=20	>=8431	8451		
male	201072	Benign prostatic hypertrophy without outflow obstruction	>=8271	<=20	8291		
female	444114	Maternal AND/OR fetal condition affecting labor AND/OR delivery	<=20	>=7957	7977		
female	4244438	Second trimester pregnancy	<=20	>=7930	7950		
female	195321	Postmenopausal bleeding	<=20	>=7715	7735		
female	4239938	First trimester pregnancy	<=20	>=7464	7484		
female	197607	Excessive and frequent menstruation	<=20	>=7064	7084		
female	194696	Dysmenorrhea	<=20	>=6991	7011		
female	200452	Disorder of female genital organs	32	6898	6930	0.0046	0.9954
female	434170	Atypical squamous cells of undetermined significance on cervical Papanicolaou smear	<=20	>=6558	6578		
female	435875	Complication of pregnancy, childbirth and/or the puerperium	24	6091	6115	0.0039	0.9961
female	435655	Gestation period, 39 weeks	<=20	>=5874	5894		
male	200962	Primary malignant neoplasm of prostate	5770	55	5825	0.9906	0.0094
female	192854	Intramural leiomyoma of uterus	<=20	>=5344	5364		
female	440795	Complication occurring during labor and delivery	<=20	>=5170	5190		
female	4132434	Gestation period, 8 weeks	<=20	>=5132	5152		
male	196738	Disorder of male genital organ	>=5087	<=20	5107		
female	442355	Gestation period, 37 weeks	<=20	>=4993	5013		
female	443871	Gestation period, 38 weeks	<=20	>=4881	4901		
female	438543	Gestation period, 36 weeks	<=20	>=4598	4618		
female	200779	Polyp of corpus uteri	<=20	>=4327	4347		
female	36716935	Acquired absence of cervix and uterus	<=20	>=4259	4279		
male	26662	Testicular hypofunction	4157	44	4201	0.9895	0.0105
female	195603	Vulval and/or perineal noninflammatory disorders	<=20	>=4136	4156		
female	36717114	Human papillomavirus deoxyribonucleic acid test positive, high risk on cervical specimen	<=20	>=4064	4084		
female	192367	Dysplasia of cervix	<=20	>=4013	4033		
female	42872398	Maternal obesity complicating pregnancy, childbirth and the puerperium, antepartum	<=20	>=3973	3993		
female	432447	Atypical glandular cells on cervical Papanicolaou smear	<=20	>=3808	3828		
female	201909	Female infertility	102	3715	3817	0.0267	0.9733
female	4094448	Pregnancy test negative	<=20	>=3783	3803		
female	4060424	Mental disorders during pregnancy, childbirth and the puerperium	<=20	>=3708	3728		
female	4279913	Primary ovarian failure	62	3591	3653	0.017	0.983
female	36712695	Suspected fetal abnormality affecting management of mother	<=20	>=3626	3646		
female	40480043	Abnormal cervical Papanicolaou smear with positive human papillomavirus deoxyribonucleic acid test	<=20	>=3578	3598		
female	441628	Menopausal and postmenopausal disorders	<=20	>=3576	3596		
female	444267	Gestation period, 35 weeks	<=20	>=3477	3497		
female	196163	Cervicitis and endocervicitis	<=20	>=3420	3440		
female	443874	Gestation period, 34 weeks	<=20	>=3363	3383		
female	432695	Post-term pregnancy	<=20	>=3199	3219		

female	40443308	Polycystic ovary syndrome	<=20	>=3080	3100		
female	436176	Miscarriage without complication	<=20	>=3060	3080		
female	433527	Endometriosis (clinical)	<=20	>=3030	3050		
female	443247	Labor and delivery complicated by fetal heart rate anomaly	<=20	>=2983	3003		
female	195770	Subserous leiomyoma of uterus	<=20	>=2981	3001		
female	4051642	Gestation period, 20 weeks	<=20	>=2961	2981		
female	432441	Finding of length of gestation	<=20	>=2931	2951		
female	442558	Gestation period, 32 weeks	<=20	>=2899	2919		
female	4171912	Abnormal cytological finding in specimen from female genital organ	<=20	>=2888	2908		
female	444098	Gestation period, 40 weeks	<=20	>=2874	2894		
female	441678	Gestation period, 33 weeks	<=20	>=2834	2854		
male	197032	Hyperplasia of prostate	2715	21	2736	0.9923	0.0077
female	45757134	Group B streptococcus infection in mother complicating childbirth	<=20	>=2698	2718		
female	195012	Intermenstrual bleeding - irregular	<=20	>=2653	2673		
female	433270	Cord entanglement without compression	<=20	>=2637	2657		
female	193739	Ovarian failure	<=20	>=2573	2593		
female	45757112	Obesity in mother complicating childbirth	<=20	>=2572	2592		
female	4097608	Gestation period, 18 weeks	<=20	>=2562	2582		
female	77619	Reduced fetal movement	<=20	>=2537	2557		
female	200461	Endometriosis of uterus	<=20	>=2522	2542		
female	444461	Gestation period, 28 weeks	<=20	>=2516	2536		
female	4181751	Gestation period, 19 weeks	<=20	>=2462	2482		
female	4113650	Noninflammatory cervical disorder	<=20	>=2421	2441		
female	40490888	Herniation of rectum into vagina	<=20	>=2416	2436		
female	45757092	Anemia in mother complicating childbirth	<=20	>=2411	2431		
female	192676	Cervical intraepithelial neoplasia grade 1	<=20	>=2346	2366		
female	4302555	Menorrhagia	<=20	>=2329	2349		
female	433864	Gestation period, 31 weeks	<=20	>=2289	2309		
female	434484	Gestation period, 30 weeks	<=20	>=2269	2289		
female	195769	Submucous leiomyoma of uterus	<=20	>=2268	2288		
female	72693	Poor fetal growth affecting management	<=20	>=2235	2255		
female	193277	Deliveries by cesarean	<=20	>=2144	2164		
female	443570	Cervicovaginal cytology: Low grade squamous intraepithelial lesion	<=20	>=2142	2162		
female	200780	Disorder of uterus	<=20	>=2140	2160		
female	438480	Abnormal glucose tolerance in mother complicating pregnancy,	<=20	>=2138	2158		
female	74104	Fetal condition affecting obstetrical care of mother	<=20	>=2130	2150		
female	444078	Inflammation of cervix	<=20	>=2118	2138		
male	196158	Disorder of penis	>=2098	<=20	2118		
female	199876	Prolapse of female genital organs	21	2080	2101	0.01	0.99
female	199067	Female pelvic inflammatory disease	<=20	>=2053	2073		
female	4095793	Female genitalia finding	<=20	>=2030	2050		
female	81636	Excessive fetal growth affecting management of mother	<=20	>=2008	2028		
female	440785	Vomiting of pregnancy	<=20	>=1983	2003		
female	4060429	Disease of the respiratory system complicating pregnancy, childbirth and/or the puerperium	<=20	>=1964	1984		
female	36683296	Polycystic ovary	<=20	>=1961	1981		
female	441364	Complication of the puerperium	<=20	>=1930	1950		
female	444417	Gestation period, 29 weeks	<=20	>=1923	1943		
female	314099	Abnormal fetal heart rate	<=20	>=1919	1939		
female	4099889	Anemia of pregnancy	<=20	>=1911	1931		
female	434089	Antepartum hemorrhage	<=20	>=1889	1909		
female	4266517	Gestation period, 13 weeks	<=20	>=1844	1864		
female	4274955	Gestation period, 22 weeks	<=20	>=1840	1860		
female	4299535	Pregnant	<=20	>=1815	1835		
female	192683	Uterovaginal prolapse	<=20	>=1813	1833		
female	40483613	Inflammatory disease of female genital structure	<=20	>=1797	1817		
female	4141640	Perimenopausal disorder	<=20	>=1781	1801		
female	4084768	Uncertain viability of pregnancy	<=20	>=1773	1793		
female	432430	Gestation period, 27 weeks	<=20	>=1763	1783		
female	4062791	Endocrine, nutritional and metabolic disease complicating pregnancy, childbirth and puerperium	<=20	>=1739	1759		
female	4024659	Gestational diabetes mellitus	<=20	>=1727	1747		
male	194997	Prostatitis	>=1672	<=20	1692		
male	196734	Disorder of prostate	>=1655	<=20	1675		
female	192978	First degree perineal tear during delivery - delivered	<=20	>=1647	1667		
female	4167493	Pregnancy-induced hypertension	<=20	>=1638	1658		
female	4336226	Gestation period, 23 weeks	<=20	>=1628	1648		
male	4320332	Hydrocele of tunica vaginalis	>=1621	<=20	1641		
female	194871	Trichomonal vulvovaginitis	<=20	>=1602	1622		

**female**	438542	Gestation less than 24 weeks	<=20	>=1598	1618		
**female**	4242241	Gestation period, 10 weeks	<=20	>=1565	1585		
**female**	4322726	Gestation less than 9 weeks	<=20	>=1563	1583		
**female**	436166	Mild hyperemesis gravidarum	<=20	>=1546	1566		
**female**	439393	Pre-eclampsia	<=20	>=1532	1552		
**female**	4049621	Gestation period, 16 weeks	<=20	>=1510	1530		
**female**	435640	Gestation period, 25 weeks	<=20	>=1495	1515		
**male**	4196960	Varicocele	>=1445	<=20	1465		
**female**	4155529	Mechanical complication of intrauterine contraceptive device	<=20	>=1393	1413		
**male**	197605	Inflammatory disorder of male genital organ	1361	42	1403	0.9701	0.0299
**female**	439081	Premenstrual tension syndrome	<=20	>=1363	1383		
**female**	4063306	Labor and delivery complication by meconium in amniotic fluid	<=20	>=1269	1289		
**female**	4217975	Normal pregnancy	<=20	>=1249	1269		
**female**	439893	Maternal obesity syndrome	<=20	>=1239	1259		
**female**	4118910	Maternal hypertension	<=20	>=1198	1218		
**female**	4248725	Gestation period, 14 weeks	<=20	>=1190	1210		
**female**	4071683	Menopausal symptom	<=20	>=1173	1193		
**male**	4270932	Pain in testicle	>=1157	<=20	1177		
**female**	435604	Viral disease in mother complicating pregnancy, childbirth AND/OR puerperium	<=20	>=1156	1176		
**female**	4194652	Pruritus of vulva	<=20	>=1121	1141		
**female**	194611	Carcinoma in situ of uterine cervix	<=20	>=1117	1137		
**female**	4283690	Gestation period, 15 weeks	<=20	>=1111	1131		
**female**	199764	Benign neoplasm of ovary	<=20	>=1110	1130		
**female**	4095475	Pregnancy test finding	<=20	>=1108	1128		
**female**	45757113	False labor before 37 completed weeks of gestation	<=20	>=1107	1127		
**female**	200051	Primary malignant neoplasm of ovary	<=20	>=1101	1121		
**female**	442781	Disorder of uterine cervix	<=20	>=1092	1112		
**female**	45757505	Perimenopausal state	<=20	>=1086	1106		
**female**	4063155	Disease of nervous system complicating pregnancy, childbirth and puerperium	<=20	>=1084	1104		
**female**	4146482	Urinary tract infection in pregnancy	<=20	>=1071	1091		
**female**	433260	Mother delivered	<=20	>=1041	1061		
**female**	432975	Trauma to perineum and/or vulva during delivery	<=20	>=1031	1051		
**female**	40480278	High risk pregnancy due to history of preterm labor	<=20	>=1001	1021		
**female**	436164	Corpus luteum cyst	<=20	>=994	1014		
**female**	440787	Drug dependence in mother complicating pregnancy, childbirth AND/OR puerperium	27	978	1005	0.0269	0.9731
